# Assessment of the Potential Role of Preoperative Dental Clearance in Total Joint Arthroplasty Optimization: A Pilot Study

**DOI:** 10.7759/cureus.68022

**Published:** 2024-08-28

**Authors:** Andrew M Jimenez, Joshua J Cook, Alec M Reihl, Nirav K Patel

**Affiliations:** 1 School of Medicine, Georgetown University, Washington D.C., USA; 2 Department of Biology, University of West Florida, Pensacola, USA; 3 Department of Orthopedics, Virginia Commonwealth University (VCU) Health, Richmond, USA; 4 Department of Hip and Knee Surgery, The Johns Hopkins University School of Medicine, Bethesda, USA

**Keywords:** total hip arthroplasty, total knee arthroplasty, total joint arthroplasty, periprosthetic joint infection, revision tja, preoperative pathway, pji, tja, dental screening

## Abstract

Aims and objectives: Periprosthetic joint infection (PJI) is a serious complication after total joint arthroplasty (TJA) and is associated with significant morbidity, mortality, and cost. This pilot study primarily aimed to investigate if preoperative dental screenings would impact the rate of PJI following TJA when compared to historical controls. Secondarily, this study aimed to evaluate the prevalence of dental pathology in patients undergoing TJA.

Methods: Charts from 103 consecutive patients undergoing primary or revision total hip arthroplasty (THA, rTHA) or total knee arthroplasty (TKA, rTKA) by a single surgeon at a single academic institution over a two-year period were reviewed and selected for inclusion. All patients were referred to a dentist for preoperative clearance using a standardized form. The rate of dental pathology before surgery, details of the dental intervention required, and any dental work performed within six months postoperatively were evaluated. The demographic and comorbidity composition of our patient population was also collected. Finally, rates of PJI following each type of TJA were obtained for demographic- and comorbidity-matched historical controls from similar study designs to examine the potential impact of preoperative dental intervention.

Results: Of the 103 patients, 31 (30.1%) were found to have preoperative dental pathology. Twenty-eight of these 31 patients (90.3%) required dental intervention prior to surgery. Based on demographic- and comorbidity-matched historical data, we expected two (95% CI (0, 6)) PJI cases for the THA group, 0 (95% CI (0, 2)) PJI cases for the TKA group, two (95% CI (0, 5)) PJI cases for the rTHA group, and two (95% CI (0, 5)) PJI cases for the rTKA group. However, in our study, there were no PJIs after any TJA up to the latest follow-up, which was unlikely for THA, rTHA, and rTKA groups given the calculated Poisson probabilities (9.39%, 15.11%, and 11.26%, respectively). Finding 0 cases was likely for the TKA group given the calculated Poisson probability of 72.61%.

Conclusions: This pilot study demonstrated that preoperative dental screening, which aims to decrease the chance of PJI due to bacteremia, may have an impact on the rate of PJI following THA, rTKA, and rTHA but not TKA based on Poisson probabilities calculated from demographic- and comorbidity-matched historical controls that lacked preoperative dental screening. For THA, rTKA, and rTHA, the Poisson probabilities of observing 0 cases of PJI postoperatively, as was the case in our study, were unlikely, suggesting that some variable in our cohort was decreasing the PJI rate for these groups. However, in the case of TKA, the Poisson probability of observing 0 cases was likely and matched the results of our study, suggesting that no variable in our cohort was affecting the PJI rate for this group. We cannot draw direct conclusions from this retrospective observational study, but the preliminary findings prompt further investigation through an appropriately controlled, blinded, multi-centered, and powered prospective randomized controlled trial.

## Introduction

Total hip arthroplasty (THA) and total knee arthroplasty (TKA) are most commonly indicated for people with osteoarthritis who have pain, limitations in activities of daily living, and have exhausted non-operative management. If primary total joint arthroplasty (TJA) fails due to infection, mechanical loosening, or instability, a revision arthroplasty is needed, exposing patients to increased levels of morbidity, limited outcomes, and a significant economic burden [1]. Revision surgeries are costly to both patients and the healthcare system, with the hospital costs of revision TKA (rTKA) alone estimated to be over $2.7 billion annually and individual surgical costs ranging from $24,027 to $38,109 [2,3]. Since periprosthetic joint infections (PJI) have become the most common indication for rTKA in 2019 and due to the increasing incidence of revision THA (rTHA) for PJI, implementing procedures that decrease PJI rates would be beneficial to both patients and hospitals [4,5]. The impact of dental pathology on PJI, due to its ability to cause bacteremia and to seed prostheses through bacteria like *Staphylococcus aureus* and *Viridans streptococci*, makes it a factor of particular interest [6-8].

A 2019 review stated that dental pathology noted in preoperative dental screens prior to TJA ranged from 8.8% to 29.4% [9]. Even so, there is currently no consensus recommendation regarding dental screening prior to TJA [10-12]. It has been suggested that patients who have poor dental hygiene (periodontitis) have higher rates of bacteremia after chewing and brushing their teeth, but it is unclear if a positive dental screening leads to increased rates of PJI or other adverse events postoperatively [7]. Nevertheless, some surgeons may inquire about any dental issues prior to performing TJA, and others suggest routine dental clearance in all patients to remove present bacterial loads [12]. This clearance can include invasive dental procedures, which involve the manipulation of the gums or perforation of the oral mucosa by removing either an active infection or a potential site for infection [13]. This practice has already consistently been used for cardiothoracic surgeons prior to heart valve surgery due to the known risk of bacterial endocarditis from streptococci [14-16]. Identifying possible risk factors for a positive dental screen may allow providers to selectively screen patients who are at high risk, thereby eliminating the costs associated with routine dental screenings in low-risk patients.

Due to the lack of evidence and conflicting data regarding preoperative dental screenings in primary, and especially revision, TJA, the primary aim of this pilot study was to investigate if preoperative dental screenings would impact the rate of PJI following TJA when compared to historical controls. Secondarily, we aimed to evaluate the prevalence of dental pathology in patients undergoing TJA.

## Materials and methods

Participants

This was a retrospective cohort study of 103 consecutive patients undergoing primary and aseptic revision hip (THA, rTHA) and knee (TKA, rTKA) arthroplasty by a single surgeon at a single academic institution over a two-year period. All patients received preoperative dental screenings in which a standardized form was used to document dental interventions, including extractions, fillings, and periodontal scaling procedures. The standard form was completed and uploaded to the medical record within a six-month period before the date of the operation. If patients were unable to use their own dentist for financial or accessibility reasons, one was arranged for them at the academic center. Exclusion criteria were patients undergoing TJA for fracture, first or single-stage revision for PJI. All patients received cefazolin perioperatively as standard antibiotic prophylaxis.

Data collection

The Institutional Review Board of Virginia Commonwealth University granted approval to access the patients’ medical records and to perform the study. A list of patients who were treated between 2018 and 2020 was acquired from a review of the institutional electronic medical record. Each patient’s medical record was examined, and the results of preoperative dental screenings were recorded. Details of any required dental intervention preoperatively, the incidence of postoperative PJI as measured by the MSIS criteria, and any dental work performed within six months postoperatively were also recorded [17]. Patient demographics including age, sex, BMI (kg/m^2), and comorbidities including diabetes mellitus, hypertension, and chronic kidney disease were recorded (Table 1), notably because of their known association with increased PJI rates [18-20].

**Table 1:** Demographics of participants Baseline patient demographics and comorbidities for each surgical group within the study cohort (THA, TKA, rTHA, and rTKA) ^1^n (%); THA: total hip arthroplasty, TKA: total knee arthroplasty, rTHA: revision total hip arthroplasty, rTKA: revision total knee arthroplasty ^2^*p<0.05, Kruskal-Wallis/Dunn’s test (numerical), Chi-squared/Fisher’s exact test (categorical) ^3^*Obesity was defined as a BMI greater than 30 kg/m^2^ TJA: total joint arthroplasty, SD: standard deviation, IQR: interquartile range, BMI: body mass index

Characteristic	THA, N = 43^1^	TKA, N = 32^1^	rTHA, N = 14^1^	rTKA, N = 14^1^	p-value^2^
Age (years)					0.13
Mean ± SD	58.12 ± 11.09	63.63 ± 7.58	61.14 ± 11.58	65.43 ± 10.07	
Median ± IQR	59.00 ± 13.00	63.00 ± 12.00	65.00 ± 14.75	65.00 ± 16.50	
Range	31.00, 76.00	52.00, 76.00	33.00, 74.00	49.00, 83.00	
Sex					0.8
Female	27 (63%)	21 (66%)	7 (50%)	8 (57%)	
Male	16 (37%)	11 (34%)	7 (50%)	6 (43%)	
BMI (kg/m^2^)					0.043*
Mean ± SD	29.09 ± 7.52	32.28 ± 4.08	29.00 ± 4.76	30.93 ± 4.62	
Median ± IQR	28.00 ± 10.00	31.00 ± 5.00	28.50 ± 6.75	30.00 ± 7.00	
Range	17.00, 52.00	25.00, 44.00	22.00, 37.00	24.00, 39.00	
Diabetes mellitus	5 (12%)	12 (38%)	2 (14%)	3 (21%)	0.060
Hypertension	18 (42%)	18 (56%)	7 (50%)	6 (43%)	0.6
Hyperlipidemia	5 (12%)	8 (25%)	1 (7.1%)	1 (7.1%)	0.3
Chronic kidney disease	3 (7.0%)	1 (3.1%)	0 (0%)	0 (0%)	0.7
Obesity^3^	19 (44%)	27 (84%)	5 (36%)	8 (57%)	0.002*
Osteoarthritis	16 (37%)	18 (56%)	3 (21%)	5 (36%)	0.13
Postoperative complications	1 (2.3%)	4 (13%)	6 (43%)	5 (36%)	<0.001*
Positive dental screen	17 (40%)	10 (31%)	3 (21%)	1 (7.1%)	0.11
Dental intervention	16 (37%)	8 (25%)	3 (21%)	1 (7.1%)	0.2
Pre-TJA extractions	10 (23%)	7 (22%)	2 (14%)	1 (7.1%)	0.6

Historical controls

To evaluate the potential impact of preoperative dental screenings on the rates of PJI following TJA, historical controls were obtained in April 2024 using the advanced search feature on PubMed. Controls were selected from the literature that evaluated the effects of antibiotic use on PJI rates. By using the control groups from these studies, we were able to isolate patient groups that were administered short-term cefazolin while avoiding variations in antibiotic type and duration following surgery that could confound PJI rates.

In addition to alignments in antibiotic treatment (i.e., standard of care cefazolin), historical datasets were chosen that best matched the demographic and comorbidity rates of our dental screening group (Table 2). Historical datasets were only chosen if the following factors were within a 15-point range of the gathered dental screening data: sex, BMI, diabetes mellitus, CKD, and obesity. A 15-point range was deemed acceptable due to its prevalence in published peer-reviewed literature.

**Table 2:** Matched historical controls (no dental screening) Demographic and comorbidity variables for matched historical controls, including age (years), sex, BMI (kg/m^2^), previous diagnosis of diabetes mellitus, hypertension, chronic kidney disease, and obesity. NA values were not included in the referenced study, and values with an asterisk (*) differed from the study cohort by at least 15 points. ^1^THA: total hip arthroplasty, TKA: total knee arthroplasty, rTHA: revision total hip arthroplasty, rTKA: revision total knee arthroplasty ^2^BMI: body mass index (kg/m^2^), DM: diabetes mellitus, HTN: hypertension, CKD: chronic kidney disease; obesity was defined as a BMI greater than 30 kg/m^2^; PJI: periprosthetic joint infection

Variable^2^	THA^1^	TKA^1^	rTHA^1^	rTKA^1^
Historical control	Dial et al. (2018) [21]	Yavuz et al. (2020) [22]	Winkler et al. (2018) [23]	Winkler et al. (2018) [23]
Age (mean)	61.50	63.40	63.50	63.80
Age (SD)	10.50	12.10	17.70	10.70
Male	64 (50.00%)	154 (30.70%)	17 (45.90%)	20 (44.40%)
Female	64 (50.00%)	348 (69.30%)	20 (54.10%)	25 (55.60%)
BMI (mean)	29.80	28.00	NA	NA
BMI (SD)	5.90	5.40	NA	NA
DM	19 (14.80%)	129 (25.70%)	8 (21.60%)	16 (35.60%)
HTN	83* (64.80%*)	296 (58.90%)	20 (54.10%)	29* (64.40%*)
CKD	10 (7.80%)	39 (7.80%)	NA	NA
Obesity	NA	NA	12 (32.40%)	31 (68.90%)
Sample size (n)	128*	502*	37*	45*
PJI	7 (5.50%)	5 (1.00%)	5 (13.50%)	7 (15.60%)

Statistical analysis

Calculations, tabulations, visualizations of descriptive statistics (means, medians, standard deviations, IQR, and ranges), and Poisson probabilities were performed in R (v.4.2.3) using RStudio (v.2024.04.2+764) and the R packages (The R Foundation for Statistical Computing, Vienna, Austria): readxl (v.1.4.2), tidyverse (v.2.0.0), gt (0.9.0), gtsummary (v.1.7.1), rcompanion (v.2.4.36), and dunn.test (v.1.3.5) [24-27]. Numerical demographic differences between the surgical groups (e.g., age, sex, BMI) were evaluated using the Kruskal-Wallis test due to the small and unequal sample sizes between the surgical groups. Furthermore, the appropriate post-hoc test (Dunn’s test) was used to determine pairwise differences. Categorical demographic differences between the surgical groups (e.g., obesity and postoperative complication rates) were evaluated using a Chi-squared test, followed up by Fisher’s exact tests with the Bonferroni correction for pairwise comparisons. The expected number of cases and associated Poisson CIs for each surgical group were calculated from historical data and then adjusted to the sample size of this cohort study. This analysis was conducted using base R programming. The Poisson probabilities and 95% CIs describe the likelihood of obtaining the PJI case numbers from this cohort when compared to expected values from closely matched historical controls. A power analysis was not performed due to the retrospective nature of this study. All R codes can be found in the Appendices section.

## Results

Participants

This study consisted of data from 103 consecutively enrolled patients, 31 (30.1%) of whom were found to have preoperative dental pathology. The mean age of this population was 61.2 years, with a range of 31 to 83 years. Additional demographic information, subset by surgical group, can be found in Table 1.

PJIs

There were no PJIs after TJA at the latest follow-up for any of the four surgical groups. However, as demonstrated by Table 3, we expected cases of PJI for the THA (n = 2), rTKA (n = 2), and rTHA (n = 2) groups after comparing the observed incidence of PJIs within our cohort to historical controls that lacked preoperative dental screens and interventions. The prevalence of hypertension was within the 15-point range for TKA and rTHA patients, but it was more varied in the control datasets for THA and rTKA. Sample size and postoperative PJI cases from these historical control groups were also obtained. The following historical control groups were selected because of their similarities to each of our surgical groups and due to their lack of preoperative dental intervention.

**Table 3:** Expected PJI cases estimated from historical controls Calculation of expected cases, Poisson probabilities, and 95% CIs for PJI adjusted for the sample sizes in this cohort study given closely matched historical controls CIs: confidence intervals, PJI: periprosthetic joint infection, THA: total hip arthroplasty, TKA: total knee arthroplasty

Surgical group	Sample size	PJI rate (%)	Expected cases	Poisson probability	95% CI lower bound	95% CI upper bound
rTHA	14	13.5	2	0.1510718	0	5
rTKA	14	15.6	2	0.1125903	0	5
THA	43	5.5	2	0.0939493	0	6
TKA	32	1.0	0	0.7261490	0	2

Preoperative dental pathology

Of the 31 patients found to have preoperative dental pathology, 28 (90.3%) required dental treatment prior to surgery. Twenty-five of the 28 (89.3%) treatments were classified as invasive, including extractions (n = 20), periodontal scaling (n = 4), and dental fillings (n = 3), with some patients receiving treatment simultaneously for multiple conditions. Three patients (2.8%) required dental treatment (one filling removal and two dental cleanings) within six months postoperatively.

THA (n = 43, expected cases = 2, 95% CI (0, 6), actual cases = 0)

After reviewing the literature, a study by Dial et al. demonstrated the most consistent procedural and compositional similarities to our dental screening group [21]. Both groups aligned in the categories of sex, BMI, diabetes, and CKD. In assessing the effectiveness of dental screenings in preventing PJI, we compared the observed incidence of PJIs within our cohort to historical rates. Based on historical data scaled to our sample size, we expected 2 (95% CI (0, 6)) cases for the THA group, with a Poisson probability of observing zero cases under these expectations of 9.39% (PJI rate = 5.5), suggesting that observing zero cases (as in our cohort) was *unlikely*.

TKA (n = 32, expected cases = 0, 95% CI (0, 2), actual cases = 0)

A study analyzing vancomycin use intraoperatively by Yavuz et al. established a control group that had a makeup resemblant to the dental screening group [22]. Values for sex, BMI, diabetes, hypertension, and CKD were within a 15-point range of the dental screening group. Based on historical data scaled to our sample size, we expected 0 (95% CI (0, 2)) cases for the TKA group, with a Poisson probability of observing zero cases under these expectations of 72.61% (PJI rate = 1.0), suggesting that observing zero cases (as in our cohort) was *likely*.

rTHA (n = 14, expected cases = 2, 95% CI (0, 5), actual cases = 0)

Although rates of PJI following revision surgeries have been less thoroughly documented in the literature than those of primary TJA, research by Winkler et al. isolated control groups for both rTHA and rTKA while also documenting important demographic factors and comorbidities for their patient population [23]. For rTHA, controls in their study closely align with our sample in regard to sex, diabetes, hypertension, and obesity [23]. Based on historical data scaled to our sample size, we expected two (95% CI (0, 5)) cases for the rTHA group, with a Poisson probability of observing zero cases under these expectations of 15.11% (PJI rate = 13.5), suggesting that observing zero cases (as in our cohort) was *unlikely*.

rTKA (n = 14, expected cases = 2, 95% CI (0, 5), actual cases = 0)

For rTKA, the comparison includes these same similarities except for rates of hypertension [23]. Based on historical data scaled to our sample size, we expected 2 (95% CI (0, 5)) cases for the rTKA group, with a Poisson probability of observing zero cases under these expectations of 11.26% (PJI rate = 15.6), suggesting that observing zero cases (as in our cohort) was *unlikely*.

Postoperative complications

In our cohort, the number of postoperative complications was statistically different among the four operative groups (p < 0.001). Specifically, the revision groups (rTHA and rTKA) had a significantly higher number of complications than primary THA (p = 0.003 and p = 0.01, respectively). Of the 32 TKA patients, four (13%) had postoperative complications that included pain, falls, stiffness, and revision surgery or required manipulation under anesthesia. Of the 14 rTHA patients, six (43%) had postoperative complications that included pain, loosening, or neuropathic pain. Of the 14 rTKA patients, five (36%) had postoperative complications that included pain, instability, foot drop, or prolonged wound care.

## Discussion

PJI is a serious complication after TJA and is associated with significant morbidity, mortality, and cost. The most significant finding from this study was that our PJI rates were lower than expected from matched historical data for rTKA, THA, and rTHA surgical groups, which may suggest that dental interventions play a role in reducing PJI for these groups. Furthermore, our study revealed the prevalence of dental pathology seen in patients prior to TJA was 30.1% (n = 103); 28 of these 31 patients (90.3%) required dental intervention prior to surgery, and three patients (2.8%) required treatment within six months postoperatively. Twenty-five (24.3%) of the patients screened in this study went on to receive invasive dental treatments preoperatively, including extraction, periodontal scaling, and/or dental fillings. The findings of this study support further investigation through an appropriately controlled, blinded, and powered randomized controlled trial.

In our study, no PJIs were observed at the latest follow-up in any of the four surgical groups, which contrasts with our expectations based on historical controls. Vuorinen et al. found a similar positivity rate for dental pathology of 29.4% in a prospective study of 952 patients who had dental screening prior to primary TJA, concluding that dental screens should be completed on all patients prior to TJA [10]. However, this study did not include revision TJA surgeries, while our findings suggest a higher probability of PJI in patients undergoing revision procedures. Fenske et al. concluded that dental referrals reduce the occurrence of early PJI, but the study was also limited by its exclusion of revision surgeries [28]. Frey et al. performed a systematic review of dental clearance before TJA and found limited evidence to support its use, with dental pathology positivity rates ranging from 8.8% to 29.4% [12]. They suggested forgoing preoperative screens due to low rates of positive findings. However, our study’s high dental positivity rate may have contributed to different conclusions than those established by Frey et al. Clinically, our results suggest that there may be potential benefits of preoperative dental screenings in reducing PJI rates, particularly for revision procedures, but there remains a need for future randomized controlled trials investigating this topic in different populations. Conversely, some studies predicted that dental intervention would spur bacteremia and increase the incidence of PJI following TJA, but a retrospective study of 591,602 patients found that dental procedures did not increase PJI risk following primary or revision TKA [29].

Our study found that 30.1% of patients had preoperative dental pathology, with 90.3% requiring treatment before surgery. Tokarski et al. found that 12% of patients required invasive dental procedures and recommended offering preoperative dental screens for "high-risk" patients [30]. However, they also noted the burden of preoperative dental treatment on patients, particularly those without access to affordable dental care. The high positivity rate in our study suggests that identifying and addressing dental pathology preoperatively could reduce PJI rates. Therefore, it is important to continue to establish significant relationships between high-risk factors and PJI to justify these interventions.

As with any retrospective study, there are limitations to our findings. Firstly, we lacked a true control group with perfectly matched demographic variables. We attempted to mitigate this by using closely matching historical controls from the literature. Secondly, our small sample size may impact the statistical power of our findings. Although our findings suggest that preoperative dental screening may have impacted PJI rates in our cohort when compared to historical controls that lacked preoperative dental screenings, we cannot conclusively claim this without conducting an appropriately controlled, blinded, and powered randomized controlled trial. Thirdly, despite our efforts to align important demographic factors and comorbidities of the historical controls with the dental screening group, variations still existed, potentially impacting our results.

Moreover, the single-surgeon, single-institution setup presents additional challenges in generalizing our findings. Potential biases in patient selection may arise due to the specific characteristics of the patient population treated by the surgeon or the institutional protocols followed, which may not be representative of the broader population. The outcomes observed could be influenced by the surgeon's individual skill, experience, and specific surgical techniques, as well as institutional factors such as postoperative care protocols and support staff expertise. These factors limit the applicability of our findings to different settings and broader patient populations.

Future randomized controlled trials should use the preliminary data gathered on our four different surgical groups to conduct a power analysis for both preoperative dental screening and non-screened groups, addressing potential ethical concerns. Furthermore, our demographic and comorbidity findings, combined with those of other studies, should be used to select variables of interest for future randomized controlled trials. These trials should be larger and more multi-centered, involving diverse populations and different healthcare settings to validate our findings and assess their generalizability. This approach may allow for the identification of possible risk factors and the implementation of a risk-based selective preoperative dental screening plan, balancing optimized care plans with healthcare expenses.

## Conclusions

This pilot study demonstrated that preoperative dental screening, which aims to decrease the chance of PJI due to bacteremia, may have an impact on the rate of PJI following THA, rTKA, and rTHA but not TKA based on Poisson probabilities calculated from demographic- and comorbidity-matched historical controls that lacked preoperative dental screening. For THA, rTKA, and rTHA, the Poisson probabilities of observing 0 cases of PJI postoperatively, as was the case in our study, were unlikely, suggesting that some variable in our cohort was decreasing the PJI rate for these groups. However, in the case of TKA, the Poisson probability of observing 0 cases was likely and matched the results of our study, suggesting that no variable in our cohort was affecting the PJI rate for this group. We cannot draw direct conclusions from this retrospective observational study, but the preliminary findings prompt further investigation through an appropriately controlled, blinded, multi-centered, and powered prospective randomized controlled trial.

## Appendices

Appendix I

A form containing the following information was completed by dentists during preoperative dental screenings:

Nirav K. Patel, MD, FRCS orthopedic surgery

Dental clearance letter

Name:____________________________________________

Date of birth:____________________

Diagnosis:________________________________________________________________________

To whom it may concern,

Our mutual patient noted above is scheduled to undergo total joint replacement surgery at VCU Health. Prior to surgery, it is important to verify the patient has had a dental examination within the last 6 months, has no current dental infection, and has no anticipation of dental care within the next six months, including restoration.

This letter is an important part of our preoperative patient evaluation, so please kindly fax this letter back to us as soon as possible.

Thank you for your assistance, and feel free to contact me should you have any concerns or questions.

Dr. Nirav K Patel, MD FRCS Assistant Professor and Orthopaedic Surgeon, VCU Health

I certify that the patient has had a dental examination within the last six months and does not have a dental infection or any significant outstanding dental issues requiring treatment.

Dentist name (please print): ______________________________________________________________

Dentist signature: ______________________________________________________________________

Date: ______________________________________________________________________________

VCU Medical Center, Department of Orthopaedic Surgery, West Hospital, 1200 East Broad Street, 9th Floor, Richmond, VA 23298-0153

Phone: (804) 828-1308 Fax: (804) 828-4762

Appendix II

# Setup

library(tidyverse) # data wrangling

library(readxl) # reading Excel files

library(gt) # tables

library(gtsummary) # summary tables

library(rcompanion) # pairwiseNominalIndependence

library(dunn.test) # dunn's test

# Raw Data

raw_data <- read_xlsx("data.xlsx")

str(raw_data)

colnames(raw_data)

# Clean Data

filter_data <-

  raw_data %>%

  select(-c(1:4, 7, 18:19, 21, 23, 28)) %>%

  slice(-c(104:107)) %>%

  mutate(

    # Replace "L" with "Left" and "R" with "Right" in the Leg column

    Leg = case_when(

      Leg == "L" ~ "Left",

      Leg == "R" ~ "Right",

      TRUE ~ Leg  # Keeps original data if it does not match "L" or "R"

    ),

    # Replace "f" with "Female" and "m" with "Male" in the Sex column

    Sex = case_when(

      Sex == "f" ~ "Female",

      Sex == "m" ~ "Male",

      TRUE ~ Sex  # Keeps original data if it does not match "f" or "m"

    ),

    # Replace "no" with "No" and "yes" with "Yes" across all columns

    across(

      where(is.character),

      ~case_when(

        . == "no" ~ "No",

        . == "yes" ~ "Yes",

        TRUE ~ .  # Keeps original data if it does not match "no" or "yes"

      )

    ),

    across(

      where(is.character),

      ~if_else(

        is.na(.), 

        "No", 

        .

      )

    )

  ) 

str(filter_data)

colnames(filter_data)

# Demographics 

filter_data %>% 

  select(-c(1, 16, 18)) %>%

  tbl_summary( #gtSummary Table

  by="Operation",

  type = list(

  c(`Age`, `BMI`) ~ 'continuous2'),

  statistic = list(all_continuous() ~ c(

    "{mean} ± {sd}",

    "{median} ± {IQR}",

    "{min}, {max}"

    )),

  digits = all_continuous() ~ 2,

  missing="ifany",

  missing_text="Missing",

  label = c(

    DM ~ "Diabetes Mellitus",

    HTN ~ "Hypertension",

    HLD ~ "Hyperlipidemia",

    CKD ~ "Chronic Kidney Disease",

    `Obesity\nBMI >=30` ~ "Obesity",

    `Osteoarthritis (OA) Medical History/Diagnosis` ~ "Osteoarthritis",

    `Dental Screen Positive?` ~ "Positive Dental Screen",

    `Intervention?` ~ "Dental Intervention"

  )

)  %>%

  add_p(

    test = list(

      all_continuous() ~ "kruskal.test",

      all_categorical() ~ "chisq.test"

      )

    ) %>%

bold_labels  %>%

add_significance_stars(thresholds=0.05)  %>%

italicize_levels() %>%

bold_p()

# Only BMI was significant; post-hoc for BMI

kruskal_test <- kruskal.test(BMI ~ Operation, data = filter_data)

if (kruskal_test$p.value < 0.05) {

  dunn_result <- dunn.test(

    x = filter_data$BMI,

    g = filter_data$Operation,

    method = "bonferroni"

  )

  print(dunn_result)

}

# Post-hoc testing for Obesity

# Contingency table

contingency_table <- table(filter_data$`Operation`, filter_data$`Obesity\nBMI >=30`)

# Perform Chi-Squared Test

chi_squared_result <- chisq.test(contingency_table)

print(chi_squared_result)

# Perform pairwise Fisher's Exact Tests with Bonferroni

pairwise_results <- pairwiseNominalIndependence(

  x = contingency_table,

  compare = "row",

  fisher = TRUE,

  chisq= TRUE,

  method = "bonferroni"

)

# Print the results

print(pairwise_results)

# Post-hoc testing for Post-Op Complications

# Contingency table

contingency_table <- table(filter_data$`Operation`, filter_data$`Post-Op Complications`)

# Perform Chi-Squared Test

chi_squared_result <- chisq.test(contingency_table)

print(chi_squared_result)

# Perform pairwise Fisher's Exact Tests with Bonferroni

pairwise_results <- pairwiseNominalIndependence(

  x = contingency_table,

  compare = "row",

  fisher = TRUE,

  chisq= TRUE,

  method = "bonferroni"

)

# Print the results

print(pairwise_results)

# Dental Screened Data

dental_data <- filter_data %>%

  select(-c(1, 8, 10, 13:18)) %>%

  rename(

    "Indication" = "Operation",

    "OA" = "Osteoarthritis (OA) Medical History/Diagnosis",

    "Obesity" = "Obesity\nBMI >=30"

    ) 

# Summarize numerical data

dental_data_summary_num <- dental_data %>%

  group_by(Indication) %>%

  summarise(

    Age_mean = mean(Age),

    Age_SD = sd(Age),

    BMI_mean = mean(BMI),

    BMI_SD = sd(BMI)

  )

# Calculate proportions and percentages for categorical data

dental_data_summary_cat <- dental_data %>%

  group_by(Indication) %>%

  summarise(

    male_percent = 100 * mean(Sex == "Male"),

    female_percent = 100 * mean(Sex == "Female"),

    DM_percent = 100 * mean(DM == "Yes"),

    HTN_percent = 100 * mean(HTN == "Yes"),

    CKD_percent = 100 * mean(CKD == "Yes"),

    Obesity_percent = 100 * mean(Obesity == "Yes"),

    OA_percent = 100 * mean(OA == "Yes")

  ) %>%

  mutate(across(starts_with("prop"), ~scales::percent(.x)))

# Combine all summaries

dental_summary <- left_join(dental_data_summary_num, dental_data_summary_cat, by = "Indication") %>%

  mutate(Type = "Dental Screening") %>%

  mutate(n = c(43, 32, 14, 14)) %>%

  mutate(PJI_percent = c(0, 0, 0, 0))

# Historical Data from literature review

historical_data <- data.frame(

  Indication = c("TKA", "THA", "rTKA", "rTHA"),

  Source = c("Yavuz (all pts given cefazolin)", "Dial (peri-op cefazolin)", "Winkler", "Winkler"),

  n_control = c(502, 128, 45, 37),

  PJI_percent = c(1, 5.5, 15.6, 13.5),

  male_percent = c(30.7, 50, 44.4, 45.9),

  female_percent = c(69.3, 50, 55.6, 54.1),

  Age_mean = c(63.4, 61.5, 63.8, 63.5),

  Age_sd = c(12.1, 10.5, 10.7, 17.7),

  BMI_mean = c(28, 29.8, NA, NA),

  BMI_sd = c(5.4, 5.9, NA, NA),

  DM_percent = c(25.6, 14.8, 35.6, 21.6),

  HTN_percent = c(58.9, 64.8, 64.4, 54.1),

  CKD_percent = c(7.8, 7.8, NA, NA),

  Obesity_percent = c(NA, NA, 68.9, 32.4),

  OA_percent = c(NA, 93, NA, NA)

)

historical_summary <- data.frame(

  Indication = c("TKA", "THA", "rTKA", "rTHA"),

  n = c(502, 128, 45, 37),

  PJI_percent = c(1, 5.5, 15.6, 13.5),

  male_percent = c(30.7, 50, 44.4, 45.9),

  female_percent = c(69.3, 50, 55.6, 54.1),

  Age_mean = c(63.4, 61.5, 63.8, 63.5),

  Age_SD = c(12.1, 10.5, 10.7, 17.7),

  BMI_mean = c(28, 29.8, NA, NA),

  BMI_SD = c(5.4, 5.9, NA, NA),

  DM_percent = c(25.6, 14.8, 35.6, 21.6),

  HTN_percent = c(58.9, 64.8, 64.4, 54.1),

  CKD_percent = c(7.8, 7.8, NA, NA),

  Obesity_percent = c(NA, NA, 68.9, 32.4),

  OA_percent = c(NA, 93, NA, NA),

  Type = c("Control", "Control", "Control", "Control")

)

str(historical_data)

# Comparison Table

comparison_summary <- bind_rows(dental_summary, historical_summary)

str(comparison_summary)

comparison_summary <- comparison_summary %>%

  mutate(Indication = factor(Indication, levels = c("TKA", "THA", "rTKA", "rTHA")))

# Pivot data for variable-based rows

long_data <- comparison_summary %>%

  pivot_longer(

    cols = c(Age_mean, Age_SD, BMI_mean, BMI_SD, male_percent, female_percent, 

             DM_percent, HTN_percent, CKD_percent, Obesity_percent, OA_percent, n, PJI_percent),

    names_to = "Variable",

    values_to = "Value"

  )

# Pivot again to spread indications into columns

wide_data <- long_data %>%

  pivot_wider(

    names_from = Indication,

    values_from = Value

  )

# Create the gt table with formatted values

wide_data %>%

  gt(groupname_col = "Type") %>%

  tab_header(

    title = "Dental Screened Data v. Historical Controls (No Dental Screen)",

    subtitle = "Numerical variables given by mean and SD, categorical by %"

  ) %>%

  cols_align(

    align = "center",

    columns = vars(TKA, THA, rTKA, rTHA)

  ) %>%

  fmt_number(

    columns = vars(TKA, THA, rTKA, rTHA),

    decimals = 2

  ) %>%

  cols_label(

    TKA = "TKA",

    THA = "THA",

    rTKA = "rTKA",

    rTHA = "rTHA"

  )

```

## Expected PJI Cases Estimated from Historical Controls

```{r}

#| echo: false

#| message: false

# Function to calculate expected cases and Poisson CI based on sample size and a given rate, 

# then computes the Poisson probability and confidence intervals. 

# dpois() and qpois() are used to calculate the Poisson probability and 

# confidence intervals, respectively.

calculate_expected_cases <- function(n, rate) {

  expected_cases <- n * rate / 100

  poisson_prob <- dpois(0, expected_cases)

  lower_bound <- qpois(0.025, expected_cases)

  upper_bound <- qpois(0.975, expected_cases)

  return(data.frame(Expected_Cases = expected_cases,

                    Poisson_Prob = poisson_prob,

                    Lower_Bound_CI = lower_bound,

                    Upper_Bound_CI = upper_bound))

}

# Filter out Historical and Dental Screening data

historical_data <- historical_summary %>%

  select(Indication, PJI_percent) 

dental_screening_data <- dental_summary %>%

  select(Indication, n)  # Sample sizes for Dental Screening

# Merge historical PJI rates with dental screening sample sizes

merged_data <- merge(dental_screening_data, historical_data, by = "Indication")

# Apply function across each row of merged data

results <- apply(merged_data, 1, function(x) calculate_expected_cases(as.numeric(x['n']), as.numeric(x['PJI_percent'])))

# Convert list of results to a dataframe

results_df <- do.call(rbind.data.frame, results)

# Round

results_df$Expected_Cases <- round(results_df$Expected_Cases)

# Combine results with merged data

final_results <- cbind(merged_data, results_df)

# Rename columns

names(final_results) <- c("Indication", "Sample_Size", "PJI_Rate", "Expected_Cases", "Poisson_Prob", "Lower_Bound_CI", "Upper_Bound_CI")

gt(final_results) # gt table

## References

[1] Bozic KJ, Kurtz SM, Lau E (2010). The epidemiology of revision total knee arthroplasty in the United States. Clin Orthop Relat Res.

[2] Bhandari M, Smith J, Miller LE, Block JE (2012). Clinical and economic burden of revision knee arthroplasty. Clin Med Insights Arthritis Musculoskelet Disord.

[3] Okafor C, Hodgkinson B, Nghiem S, Vertullo C, Byrnes J (2021). Cost of septic and aseptic revision total knee arthroplasty: a systematic review. BMC Musculoskelet Disord.

[4] Upfill-Brown A, Hsiue PP, Sekimura T (2022). Epidemiology of revision total knee arthroplasty in the United States, 2012 to 2019. Arthroplast Today.

[5] Upfill-Brown A, Hsiue PP, Sekimura T, Patel JN, Adamson M, Stavrakis AI (2021). Instability is the most common indication for revision hip arthroplasty in the United States: national trends from 2012 to 2018. Arthroplast Today.

[6] Alamanda VK, Springer BD (2018). Perioperative and modifiable risk factors for periprosthetic joint infections (PJI) and recommended guidelines. Curr Rev Musculoskelet Med.

[7] Forner L, Larsen T, Kilian M, Holmstrup P (2006). Incidence of bacteremia after chewing, tooth brushing and scaling in individuals with periodontal inflammation. J Clin Periodontol.

[8] Danilkowicz RM, Lachiewicz AM, Lorenzana DJ, Barton KD, Lachiewicz PF (2021). Prosthetic joint infection after dental work: is the correct prophylaxis being prescribed? A systematic review. Arthroplast Today.

[9] Jafari SM, Coyle C, Mortazavi SM, Sharkey PF, Parvizi J (2010). Revision hip arthroplasty: infection is the most common cause of failure. Clin Orthop Relat Res.

[10] Vuorinen M, Mäkinen T, Rantasalo M, Leskinen J, Välimaa H, Huotari K (2019). Incidence and risk factors for dental pathology in patients planned for elective total hip or knee arthroplasty. Scand J Surg.

[11] Lampley A, Huang RC, Arnold WV, Parvizi J (2014). Total joint arthroplasty: should patients have preoperative dental clearance?. J Arthroplasty.

[12] Frey C, Navarro SM, Blackwell T, Lidner C, Del Schutte H Jr (2019). Impact of dental clearance on total joint arthroplasty: a systematic review. World J Orthop.

[13] Perez-Chaparro PJ, Meuric V, De Mello G, Bonnaure-Mallet M (2011). Bacteremia of oral origin (Article in French). Rev Stomatol Chir Maxillofac.

[14] Rao NR, Treister N, Axtell A (2020). Preoperative dental screening prior to cardiac valve surgery and 90-day postoperative mortality. J Card Surg.

[15] Baddour LM, Wilson WR, Bayer AS (2015). Infective endocarditis in adults: diagnosis, antimicrobial therapy, and management of complications: a scientific statement for healthcare professionals from the American Heart Association. Circulation.

[16] Ambrosioni J, Hernandez-Meneses M, Téllez A (2017). The changing epidemiology of infective endocarditis in the twenty-first century. Curr Infect Dis Rep.

[17] Parvizi J, Tan TL, Goswami K, Higuera C, Della Valle C, Chen AF, Shohat N (2018). The 2018 definition of periprosthetic hip and knee infection: an evidence-based and validated criteria. J Arthroplasty.

[18] Eka A, Chen AF (2015). Patient-related medical risk factors for periprosthetic joint infection of the hip and knee. Ann Transl Med.

[19] Bae KJ, Chae YJ, Jung SJ, Gong HS (2022). Incidence and risk factors for periprosthetic joint infection: a common data model analysis. Jt Dis Relat Surg.

[20] (2020). Risk factors for periprosthetic joint infection following primary total hip arthroplasty: a 15-year, population-based cohort study. J Bone Joint Surg Am.

[21] Dial BL, Lampley AJ, Green CL, Hallows R (2018). Intrawound vancomycin powder in primary total hip arthroplasty increases rate of sterile wound complications. Hip Pelvis.

[22] Yavuz IA, Oken OF, Yildirim AO, Inci F, Ceyhan E, Gurhan U (2020). No effect of vancomycin powder to prevent infection in primary total knee arthroplasty: a retrospective review of 976 cases. Knee Surg Sports Traumatol Arthrosc.

[23] Winkler C, Dennison J, Wooldridge A, Larumbe E, Caroom C, Jenkins M, Brindley G (2018). Do local antibiotics reduce periprosthetic joint infections? A retrospective review of 744 cases. J Clin Orthop Trauma.

[24] (2024). Read Excel Files [R Package readxl Version 1.4.3]. https://cran.r-project.org/web/packages/readxl/index.html.

[25] Wickham H, Averick M, Bryan J (2019). Welcome to the Tidyverse. J Open Source Softw.

[26] Iannone R, Cheng J, Schloerke B (2024). gt: Easily Create Presentation-Ready Display Tables. https://gt.rstudio.com/.

[27] Sjoberg DD, Whiting K, Curry M, Lavery J A, Larmarange J (2021). Reproducible summary tables with the gtsummary package. The R Journal.

[28] Fenske F, Kujat B, Krause L (2024). Preoperative dental screening can reduce periprosthetic infections of hip and knee endoprostheses in the first month after surgery: results of a cohort study. Infection.

[29] Park HJ, Koh K, Choi YJ, Suh DH, D'Lima D, Kim JG (2024). Is prophylactic antibiotic use necessary before dental procedures in primary and revision TKA? A propensity score-matched, large-database study. Clin Orthop Relat Res.

[30] Tokarski AT, Patel RG, Parvizi J, Deirmengian GK (2014). Dental clearance prior to elective arthroplasty may not be needed for everyone. J Arthroplasty.

